# 

**Published:** 1955-09

**Authors:** 


### THE MEDICAL JOURNAL OF THE SOUTH-WEST

*Incorporating*
*The Bristol Medico-Chirurgical Journal*

ARMED FORCES
SEP 12 1955
MEDICAL LIBRARY

*September 1955*

VOL. 70 (v). No. 257

Price 4/-

